# Molecular phylogeny of ocelloid-bearing dinoflagellates (Warnowiaceae) as inferred from SSU and LSU rDNA sequences

**DOI:** 10.1186/1471-2148-9-116

**Published:** 2009-05-25

**Authors:** Mona Hoppenrath, Tsvetan R Bachvaroff, Sara M Handy, Charles F Delwiche, Brian S Leander

**Affiliations:** 1 Departments of Botany and Zoology, University of British Columbia, 6270 University Boulevard, Vancouver, BC, V6T 1Z4, Canada; 2 Smithsonian Environmental Research Center, Edgewater, MD 21037-0028, USA; 3 Department of Cell Biology and Molecular Genetics, University of Maryland, College Park, MD 20742-4407, USA; 4 Current address : Forschungsinstitut Senckenberg, Deutsches Zentrum für Marine Biodiversitätsforschung (DZMB), Südstrand 44, D-26382 Wilhelmshaven, Germany

## Abstract

**Background:**

Dinoflagellates represent a major lineage of unicellular eukaryotes with unparalleled diversity and complexity in morphological features. The monophyly of dinoflagellates has been convincingly demonstrated, but the interrelationships among dinoflagellate lineages still remain largely unresolved. Warnowiid dinoflagellates are among the most remarkable eukaryotes known because of their possession of highly elaborate ultrastructural systems: pistons, nematocysts, and ocelloids. Complex organelles like these are evolutionary innovations found only in a few athecate dinoflagellates. Moreover, the taxonomy of warnowiids is extremely confusing and inferences about the evolutionary history of this lineage are mired by the absence of molecular phylogenetic data from any member of the group. In this study, we provide the first molecular phylogenetic data for warnowiids and couple them with a review of warnowiid morphological features in order to formulate a hypothetical framework for understanding character evolution within the group. These data also enabled us to evaluate the evolutionary relationship(s) between warnowiids and the other group of dinoflagellates with complex organelles: polykrikoids.

**Results:**

Molecular phylogenetic analyses of SSU and LSU rDNA sequences demonstrated that warnowiids form a well-supported clade that falls within the more inclusive *Gymnodinium *sensu stricto clade. These data also confirmed that polykrikoids are members of the *Gymnodinium *sensu stricto clade as well; however, a specific sister relationship between the warnowiid clade and the polykrikoid clade was unresolved in all of our analyses. Nonetheless, the new DNA sequences from different isolates of warnowiids provided organismal anchors for several previously unidentified sequences derived from environmental DNA surveys of marine biodiversity.

**Conclusion:**

Comparative morphological data and molecular phylogenetic data demonstrate that the polykrikoid and the warnowiid clade are closely related to each other, but the precise branching order within the *Gymnodinium *sensu stricto clade remains unresolved. We regard the ocelloid as the best synapomorphy for warnowiids and infer that the most recent common ancestor of polykrikoids and warnowiids possessed both nematocysts and photosynthetic plastids that were subsequently lost during the early evolution of warnowiids. Our summary of species and genus concepts in warnowiids demonstrate that the systematics of this poorly understood group is highly problematic and a comprehensive revision is needed.

## Background

Dinoflagellates represent a major lineage of unicellular eukaryotes with unparalleled diversity and complexity in morphological features, molecular processes, nutritional modes and symbioses with distantly related organisms [1-3]. The ecological importance of dinoflagellates is also extraordinary; members of the group play key roles as marine primary producers, coral reef zooxanthellae, and (micro)consumers in aquatic communities around the globe. The monophyly of dinoflagellates and their relationship to other alveolate taxa – particularly apicomplexans and ciliates – have been convincingly demonstrated with congruent molecular phylogenetic data [e.g., [1,4-9]]. However, the interrelationships among dinoflagellate lineages still remain largely unresolved, especially near the phylogenetic backbone of the group [e.g., [10-12]]. Although significant events in the evolutionary radiation of dinoflagellate diversity have been inferred from comparative analyses of morphological characters alone [e.g., [13]], the coupling of these data with molecular phylogenetic data, including environmental DNA surveys of biodiversity, has more robustly demonstrated delimitations between 'species' and the monophyly of several dinoflagellate subgroups [e.g., [10,11,14]].

### Brief history of athecate dinoflagellate systematics

Understanding the phylogenetic relationships of athecate (syn. unarmored or naked) dinoflagellates has been problematic for more than a century, because of difficulties in identifying reliable morphological characters with light microscopy. For instance, overlapping and ambiguous criteria, such as episome dimensions and the displacement of the cingulum, have been used in the past to distinguish genera from one another. Improved methods incorporating both molecular and morphological data have been used to re-investigate the type species of different athecate genera that have long been recognized to be polyphyletic, such as *Gymnodinium *Stein, *Gyrodinium *Kofoid et Swezy, and *Amphidinium *Claparède et Lachmann [10,15-18]. More precise re-definitions of these genera have caused many of the species that were formerly assigned to them to be considered "sensu lato taxa". Accordingly, several new genera have been established over the past decade to accommodate these newly recognized lineages, such as *Akashiwo *Hansen and Moestrup,*Karenia *Hansen and Moestrup, *Karlodinium *Larsen, *Takayama *de Salas, Bolch, Botes et Hallegraeff, *Togula *Flø Jørgensen, Murray et Daugbjerg, *Prosoaulax *Calado et Moestrup, and *Apicoporus *Sparmann, Leander et Hoppenrath [10,19-22].

Apical surface structures found in athecate dinoflagellates using electron microscopy, such as 'acrobases' (apical grooves) and apical pores with hook-like protrusions, have turned out to be phylogenetically meaningful features that are consistent with more natural classification systems [10,19,22-24]. Moreover, variable features of chloroplasts (or more generally 'plastids') can be diagnostic at both the generic level, such as *Karenia *and *Lepidodinium*, and the species level, such as within *Gymnodinium *and *Polykrikos *Bütschli [e.g., [25-27]]. Characters like the formation of pseudocolonies and the possession of complex organelles are evolutionary innovations found only in a few athecate dinoflagellates, namely polykrikoids and warnowiids [27-29]. Although the value of these characteristics for establishing robust phylogenetic hypotheses is expected to be high, molecular phylogenetic data are still unavailable for most of these lineages. This is mainly due to the fact that polykrikoids and warnowiids are uncultivated and difficult to both find and isolate from natural marine samples [27,28,30].

### Complex organelles in athecate dinoflagellates

**Figure 1:**
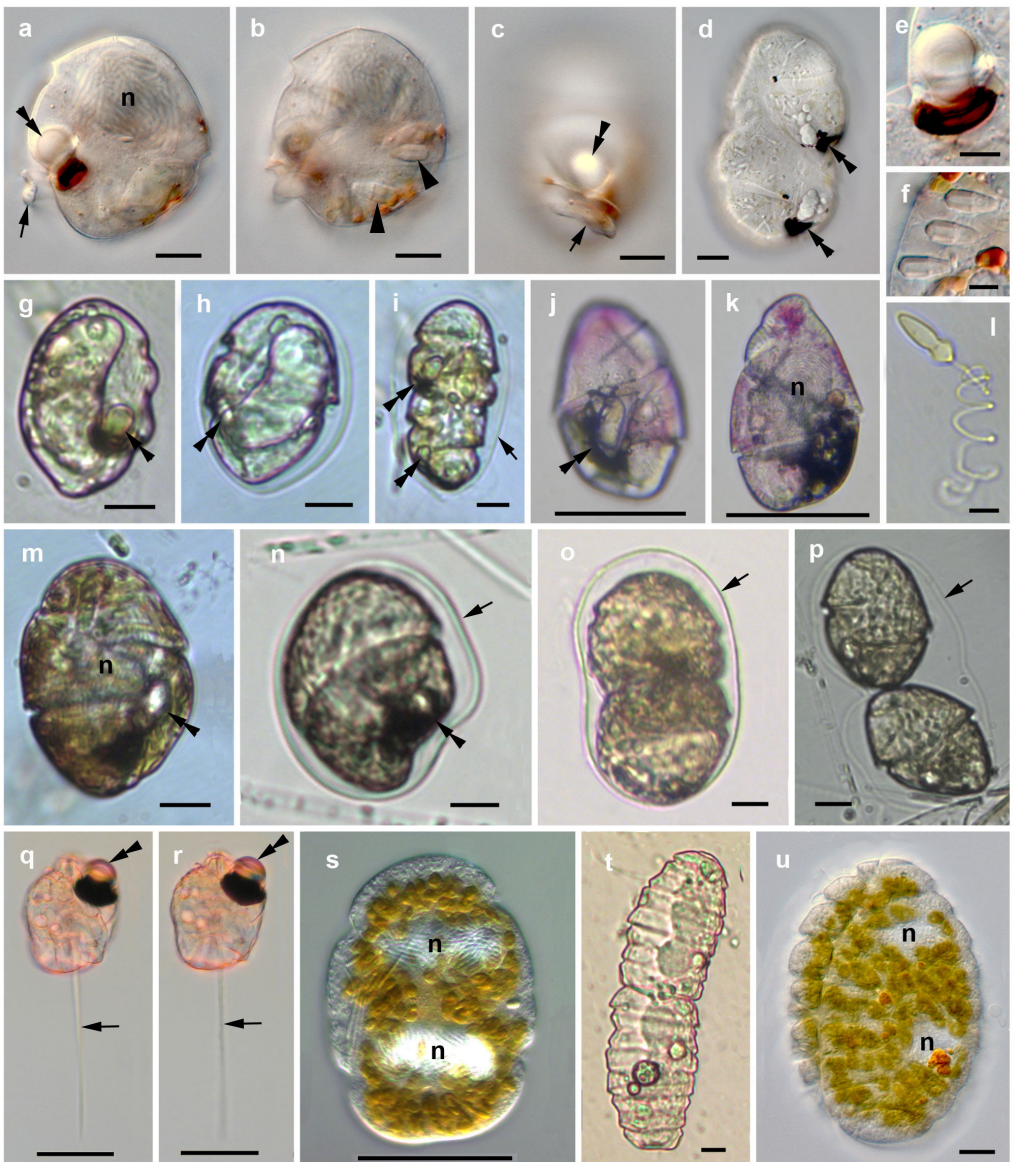
**Light micrographs of the investigated warnowiid and polykrikoid taxa**. **(a)-(f) **Images representing isolates 1 and 2 of '*Proterythropsis*' sp. **(a) **Lateral view, median focus showing the large nucleus (n), the ocelloid (double arrowhead), and the posterior cell 'extension' (arrow). **(b) **Lateral view, showing the nematocysts (arrowheads). **(c) **Left ventral view showing the posterior cell 'extension' (arrow) and the ocelloid (double arrowhead). **(d) **Dividing cell with partly reassembled ocelloids/hyalosomes (double arrowheads) in the developing daughter cells. **(e) **Ocelloid. **(f) **Nematocysts. **(g)-(i) **Images representing the isolate of *Warnowia *sp. (British Columbia). (g) Lateral view of a free swimming cell showing the ocelloid (double arrowhead). **(h) **Lateral view of a cell in a hyaline cyst (arrow) showing the ocelloid (double arrowhead). **(i) **Dividing cell in a hyaline cyst (arrow) showing the ocelloids (double arrowheads). **(j)-(k) **Images showing the isolate of "*Warnowia *sp." (Florida) used for single cell PCR. **(j) **Ventral view, surface focus, showing the ocelloid (double arrowhead). **(k) **Mid cell focus showing the large nucleus (n). **(l) **An extruded nematocyst of *Polykrikos kofoidii*. (m)-(p) Images representing the two isolates of '*Nematodinium*' sp. **(m) **Lateral to ventral view of a free swimming cell showing the large nucleus (n), the ocelloid (double arrowhead), and the brownish chloroplast color. **(n) **Lateral view of a cell in a hyaline cyst (arrow) showing the ocelloid (double arrowhead). **(o) **Dividing cell in a hyaline cyst (arrow). **(p) **Image showing recently divided daughter cells within the hyaline cyst (arrow). **(q)-(r) **Images of *Erythropsidinium *sp. showing the ocelloid (double arrowheads), and the piston (arrows). **(s) **Image representing the isolate of '*Pheopolykrikos*' *hartmannii *showing the two large nuclei in the pseudo-colony. **(t) **Image representing the isolate of *Polykrikos kofoidii *undergoing division of the pseudo-colony. **(u) **Images of *Polykrikos lebourae *showing the two nuclei within the pseudo-colony. Scale bars 10 μm in (a)-(d), (g)-(i), (l)-(p), (t), (u), 20 μm in (j), (k), (q)-(s), 5 μm in (e), (f).

Polykrikoids and warnowiids are among the most remarkable eukaryotes on the planet because of their possession of highly elaborate ultrastructural systems: pistons, nematocysts, nematocyst-taeniocyst complexes and ocelloids [29] (Figure 1). A piston is a relatively long posterior 'tentacle' with an exaggerated capacity for rapid and repeated contraction and is, so far, known only in two genera (*Erythropsidinium *and *Greuetodinum*) [29]. The function of the piston remains unknown. Dinoflagellate nematocysts are found only in some polykrikoids and warnowiids, and are composed of one or several extrusive filaments. The detailed morphology of the nematocysts differs in the two groups [29] (see also Figures 1f and 1l), and in *Polykrikos*, the nematocysts are linked to additional extrusive organelles called 'taeniocysts' [29,31]. The nematocyst-taeniocyst complex of *Polykrikos *species is a synapomorphy for the genus, but the presence of nematocysts in warnowiids suggests that these two lineages are closely related (i.e., dinoflagellate nematocysts are homologous) [27,28]. Another formal possibility is that nematocysts evolved twice independently within athecate dinoflagellates: once in polykrikoids and once in warnowiids. This scenario is not unprecedented, because cnidarians, which are very distantly related to dinoflagellates, also possess different kinds of cnidae (e.g., nematocysts and spirocysts) within cells called 'cnidocytes' that presumably evolved independently from those found in athecate dinoflagellates. However, a scenario involving kleptocnidae is also possible, whereby nematocysts were horizontally transferred between cnidarians and dinoflagellates during evolutionary history [32]. Regardless, molecular phylogenetic data from warnowiid species are required to shed additional light onto these hypotheses, which was one of the main aims of this study.

Perhaps the most complex organelle found in any dinoflagellate so far happens to be synapomorphic for warnowiids, namely a distinctive multilayered photoreceptor called an 'ocelloid' [29] (see also Figure 1e). Ocelloids are composed entirely of subcellular components and are highly reminiscent in general organization to the multicellular camera eyes that evolved independently in several different lineages of metazoans (e.g. cubozoans, scallops, cephalopods and vertebrates) [33]. The complexity of these subcellular systems is so distinctive that ocelloids have been described as "the most extraordinarily sophisticated differentiations of grouped structures for a single function in protists" [13]. Ocelloids are comprised of two main components: a hyalosome and a melanosome [29]. The translucent haylosome consists of a layered cornea-like structure and a lens-like inclusion that are bounded at the base by iris-like constriction rings; the melanosome is a highly ordered and pigmented retina-like body that is separated from the hyalosome by a seawater chamber. In fact, the melanosome (syn. pigment cup or retinal body) appears to be a highly derived plastid with thylacoids that can be recognized when the melanosome becomes relatively unordered during cell division and daughter cell formation; by contrast, the hyalosome appears to be synthesized by the cell and is disassembled during cell division before being reassembled in each daughter cell [[29]; this study]. The structural details of ocelloids are specific to different warnowiid lineages (e.g. the number of constriction rings and the position of the ocelloid in the dinoflagellate cell) [29], and this variation provides important insights into the evolutionary history of the clade.

### Other general features of warnowiids

Although most are heterotrophic, three of the about 40 described species of polykrikoids and warnowiids are photosynthetic, namely *Pheopolykrikos hartmannii *(Zimmermann) Matsuoka et Fukuyo, *Polykrikos lebourae *Herdman, and *Nematodinium armatum *(Dogiel) Kofoid et Swezy [e.g., [27,28,34,35]]. The cingulum in warnowiids is always displaced and encircles the cells at least once and sometimes more than twice. Some warnowiids produce mucoid hyalin cysts (Figures 1h, n), probably vegetative division cysts, as shown in our Figures 1i, o and 1p. The taxonomy of warnowiids is poorly understood and confusing, and many of the species described by Kofoid and Swezy [36] are probably conspecific. Some reports indicate that the structure, color and position of the ocelloid can change during the course of cell division and development [37], and that these ocelloid features are unreliable taxonomic criteria for delimiting species and genera [37,38]. Accordingly, different authors have adopted different classification systems when discussing the group (Table 1) [6,39-41].

**Table 1:** Systematics of warnowiid genera (Warnowiaceae Lindemann 1928).

Fensome et al. 1993 [6]	Sournia 1986 [39]	Steidinger & Tangen 1997 [40]	Gómez 2005 [41]
*Warnowia *Lindemann 1928 (type genus)	*Warnowia*	*Warnowia*	*Warnowia*
Syn.: *Pouchetia *Schütt 1895	Syn.: *Pouchetia*	Syn.: *Pouchetia*	Syn.: *Pouchetia*
	Syn.: *Protopsis*	Syn.: *Protopsis*	Syn.: *Protopsis*
	Syn.: ? *Proterythropsis *partim		
*Erythropsidinium *Silva 1960	*Erythropsidinium*	*Erythropsidinium*	*Erythropsidinium*
Syn.: *Erythropsis *Hertwig 1884	Syn.: *Erythropsis*	Syn.: *Erythropsis*	Syn.: *Erythropsis*
	Syn.: *Pouchetia *partim		Syn.: *Pouchetia *partim
*Greuetodinium *Loebl. III 1980	*Greuetodinium*		taxon of doubtful validity
Syn.: *Leucopsis *Greuet 1968	Syn.: *Leucopsis*		taxon of doubtful validity
*Nematodinium *Kof. et Sw. 1921	*Nematodinium*	*Nematodinium*	*Nematodinium*
	Syn.: *Nematopsides*	Syn.: *Nematopsides*	
		Syn.: *Pouchetia*	Syn.: *Pouchetia*
*Nematopsides *Greuet 1973			taxon of doubtful validity
*Proterythropsis *Kof. et Sw. in Kofoid 1920			*Proterythropsis *Kof. et Sw. 1921
*Protopsis *Kof. et Sw. 1921			taxon of doubtful validity

Loebl. = Loeblich; Kof. et Sw. = Kofoid et Swezy

In order to more clearly outline the taxonomic challenges associated with understanding warnowiid diversity, we have summarized the morphological features described for each warnowiid genus in Table 2[24,29,30,34-38,42-53]. Currently, *Erythropsidinium *and *Greuetodinium *are characterized by having a piston, and *Greuetodinium *is separated from *Erythropsidinium *by possessing the only ocelloid of the composite type (multiplication of 'lenses') at the anterior end of the cell; *Proterythropsis *also has a projection off of the posterior end of the cell, but it is immobile and called a 'posterior extension'. Nematocysts have only been found in *Nematodinium *and *Proterythropsis*. Although *Warnowia *Lindemann lacks plastids, nematocysts, a piston and a posterior extension, a relatively broad spectrum of cell morphologies associated with the acrobase, cingulum, sulcus, and position of the ocelloid has been described within this genus; thus, *Warnowia *probably represents an artificial assemblage of species. Nonetheless, inferences about the evolutionary history of warnowiids, in general, are mired by the absence of molecular phylogenetic data from any member of the group.

**Table 2:** Morphological features of warnowiid dinoflagellate genera.

	*Nematodinium*^1^	*Proterythropsis*^2^	*Warnowia*^3^	*Erythropsidinium*^4^	*Greuetodinium*^5^
**ocelloid**					
position in the cell	posterior	posterior	~cell middle or posterior	anterior	Anterior
directed	ventral	ventral	ventral to anteriorly	anteriorly	Anteriorly
relative size	small	small	medium	large	very large
# constriction rings	1	?	2	3	?
'pigment ring'	band, continuity with upper retinal body	?	ring, isolated from retinal body	ring, totally independent from retinal body	?
# per cell	1	1	1	1 (or 2*)	~15 lenses
	integrate type	integrate type	integrate type	integrate type	composite type
**nematocysts**	yes (and no, see Hulburt 1957	yes (and no, see Kofoid & Swezy 1921)	no	no (but see Hertwig 1884)	no
**feeding apparatus**	?	?	?	stomopharyngian complex	?
**chloroplasts**	yes and no	no	no (but see Hulburt 1957)	no	no
**nucleus position**	upper cell half	upper cell half	middle or upper cell half	right upper cell half	median upper cell half
**acrobase**	outward left spiral, 1 turn (or little more) = loop in *Gymn. s.s.*	outward left spiral, 1 turn (or little more) = loop in *Gymn. s.s.*	inward left spiral, 1.5–2.0 turns, some species plus outward left spiral	angled left 'spiral', 2 turns	? (ventral below the cingulum)
**cingulum**	~1.5–2.25 turns	~1.25–2.0 turns	~1.0–2.5 turns	1.5 turns	1 turn, steep descending dorsally
**sulcus**	< 1–2.0 turns	< 1 turn	0.5–1.75 turns	straight	nearly one turn
**cell 'extension' **(tentacle)	no	yes	no	no	? maybe
**piston**	non	non	non	1 (seldom 2*)	1
terminal stylet	-	-	-	sometimes	?

^1 ^[24,29,34-37,42,43,47,49]; ^2 ^[36,38], present study;^3 ^[24,29,36,37,45]; ^4 ^[24,29,30,36,37,44-46,48,50-52]; ^5 ^[53]; * [30]; ? = no data; *Gymn. s.s*. = *Gymnodinium *sensu stricto.

In this study, we provide the first molecular phylogenetic data for warnowiids: small subunit (SSU) rDNA sequences were obtained from two species of '*Warnowia*' (one from British Columbia and one from Florida), two isolates of '*Nematodinium *sp.' (both from British Columbia), and two isolates of '*Proterythropsis *sp.' (both from British Columbia); partial large subunit (LSU) rDNA sequences were obtained from one species of '*Nematodinium*' (from British Columbia) and one species of *Warnowia *(from British Columbia). Moreover, we obtained partial LSU rDNA sequences from three polykrikoid species: *Polykrikos kofoidii *(from British Columbia), *Polykrikos lebourae *(from British Columbia), and *'Pheopolykrikos' hartmannii *(from Maryland). These molecular phylogenetic data were coupled with a review of warnowiid morphological data (Table 2) in order to formulate a hypothetical framework for understanding character evolution within the group.

## Methods

### Collection of organisms and light microscopy

Near surface plankton samples were collected in the morning hours with a small net (mesh-size 20 μm) at the docks of the Bamfield Marine Sciences Center, Vancouver Island, BC (48°50.0' N, 125°8.0' W) in June 2005, April 2006, April 2007 and May 2007. Immediately after sampling, single cells of the species were identified at 40× to 250× magnifications and isolated from the mixed plankton sample by micropipetting for the preparations described below. Cells were observed directly and micromanipulated with a Leica DMIL inverted microscope connected to a PixeLink Megapixel color digital camera. For DIC light microscopy, micropipetted cells were placed on a glass specimen slide and covered with a cover slip. Images were produced directly with either the PixeLink Megapixel color digital camera or a Zeiss Axioplan 2 imaging microscope connected to a Leica DC500 color digital camera. Sand samples containing *Polykrikos lebourae *were collected with a spoon during low tide at Centennial Beach, Boundary Bay, BC (49°0.0' N, 123°8.0' W) in May 2007. The sand samples were transported directly to the laboratory, and the flagellates were separated from the sand by extraction through a fine filter (mesh size 45 μm) using melting seawater-ice [54]. The flagellates accumulated in a Petri dish beneath the filter and were then identified at 40× to 250× magnifications. Cells were isolated by micropipetting for the preparations described below.

Samples containing *Erythropsidinium *and an unidentified "*Warnowia *sp." (Florida) were collected from the Gulf Stream off of Ft. Pierce, Florida (N27° 28.25' and W79° 53.62') on August 28, 2007. *Pheopolykrikos hartmannii *was collected from the Rhode River, MD at the Smithsonian Environmental Research Center (SERC) dock (N38° 53.1' W 76° 32.5') on July 31, 2007. A horizontal plankton tow was taken from the surface layer using a net with a mesh-size of 35 μm. Samples were held at ambient temperature and transported to the lab. The sample was screened using a 250 μm-mesh Nitex sieve to remove large zooplankton, and diluted with seawater to enhance viability during transport. Cells were visualized through a dissecting microscope and individually picked using drawn glass tubing and mouth aspiration. Each cell was washed six times with 0.2 μm filtered station water, photographed, placed into a sterile 1.5-mL microfuge tube containing 40 μL of lysis buffer, amended with Igepal instead of nonidet P40, and frozen at -80°C [55].

### Scanning electron microscopy

A mixed-extraction sample containing *'Proterythropsis' *sp. was fixed with OsO_4 _for 30 min at room temperature. Cells were transferred onto a 5-μm polycarbonate membrane filter (Corning Separations Div., Acton, MA), washed with distilled water, dehydrated with a graded series of ethanol and critical point dried with CO_2_. The filter was mounted on a stub, sputter-coated with gold and viewed under a Hitachi S4700 Scanning Electron Microscope.

### DNA extraction, PCR amplification and sequencing

Cells collected in British Columbia were manually isolated and washed three times in f/2-medium. Three different methods for DNA extraction were used over the years. (1) Collected cells were placed directly into 400 μL CTAB extraction buffer (1.12 g Tris, 8.18 g NaCl, 0.74 g EDTA, 2 g CTAB, 2 g Polyvinylpyrolidone, 0.2 mL 2-mercaptoethanol in 100 ml water) in 1.5 mL Eppendorf tube. The tube was placed in a heat-block and incubated at 63°C for 20 min with several vigorous shakes in between. After separation with chloroform:isoamyl alcohol (24:1), the aqueous phase was precipitated in 70% ethanol. The dry DNA pellets were stored in the freezer and transported to the University of British Columbia on ice. Distilled water was added to each sample prior to PCR. (2) Genomic DNA was extracted by making a final washing step in distilled water, and the osmotically disrupted cells were used directly for PCR. (3) Genomic DNA was extracted using the MasterPure Complete DNA and RNA Purification Kit (EPICENTRE, Madison, WI, USA). The small subunit rDNA sequence was PCR amplified using puReTaq Ready-to-go PCR beads (GE Healthcare, Quebec, Canada), with an error rate of 1 per 20,000–40,000 bases, and universal eukaryotic primers reported previously [[56]; Table 3]. The large subunit rDNA sequence was also PCR amplified using puReTaq Ready-to-go PCR beads and D1R-R2 primers published by Scholin et al. [57] and Yamaguchi et al. [58] (Table 3). Information about the date of collection, number of isolated cells, method of DNA extraction and primer combination for each DNA sequence is shown in Table 4 [GenBank accession codes FJ947036–FJ947046]. PCR products of the expected size were gel isolated and cloned into pCR2.1 vector using a TOPO TA cloning kit (Invitrogen Corporation, CA, USA). One clone for each species was completely sequenced with ABI big-dye reaction mix using both vector primers and two internal primers oriented in both directions.

**Table 3:** Primers used for PCR.

Primer name	sequence 5'-3'	Target	Citation
PF1	GCGCTACCTGGTTGATCCTGCC	SSU	[56] (modified)
R4	GATCCTTCTGCAGGTTCACCTAC	SSU	[56] (modified)
D1R	ACCCGCTGAATTTAAGCATA	LSU	[57]
R2	ATTCGGCAGGTGAGTTGTTAC	LSU	[58]
			
Euk A	AACCTGGTTGATCCTGCCAGT	SSU	[59] (modified)
Euk B	GATCCWTCTGCAGGTTCACCTAC	SSU	[59]
Dino1662 F	CCGATTGAGTGWTCCGGTGAATAA	SSU	[60]
SR 4	AGGGCAAGTCTGGTGCCAG	SSU	[61]
SR 9	AACTAAGAACGGCCATGCAC	SSU	[61]
SR 8	GGATTGACAGATTGAKAGCT	SSU	[63]
SR 12	CCTTCCGCAGGTTCACCTAC	SSU	[58]
Dino R	TTATTCACCGGAWCACTCAATCGG	SSU	this manuscript
25 F1	CCGCTGAATTTAAGCATAT	LSU	[62]
25 R1	CTTGGTCCGTGTTTCAAGAC	LSU	[61]
LSU D3A	GACCCGTCTTGAAACACGGA	LSU	[63]
LSU R2	ATTCGGCAGGTGAGTTGTTAC	LSU	[58]
DLSU	CTGTTAAAATGAACCAACACCYTTT	LSU	this manuscript

**Table 4:** Date of collection, number of isolated cells, method of DNA extraction and primer combination for each DNA sequence reported in this study.

Species	isolation date	# of cells	DNA extraction	primer combination
**SSU**				
'*Proterythropsis*' sp. 1	24. June 2005	12	CTAB	PF1-R4
'*Proterythropsis*' sp. 2	25. June 2005	27	CTAB	PF1-R4
'*Nematodinium*' sp. 1	27. April 2006	2	Distilled water	PF1-R4
'*Nematodinium*' sp. 2	28. April 2006	3	Distilled water	PF1-R4
*Warnowia *sp. (BC)	02. May 2007	3	Kit	PF1-R4
"*Warnowia *sp." (Florida)	28. Aug. 2007	1	Sonication	EukA-EukB
**LSU**				
'*Nematodinium*' sp.	28. April 2007	3	Kit	D1R-R2
*Polykrikos kofoidii*	28. April 2007	28	Kit	D1R-R2
*Warnowia *sp. (BC)	02. May 2007	3	Kit	D1R-R2
*Polykrikos lebourae*	18. May 2007	8	Kit	D1R-R2
'*Pheopolyk*.' *hartmannii*	31. July 2007	1	Sonication	Dino1662F-25R1
				25F1-LSUR2

"1" and "2" after species names refer to different isolates; primers sequences are listed in Table 3.

Warnowiid cells collected from the east coast of North America were frozen in microfuge tubes before being thawed and sonicated using a probe tipped sonicator (Heat Systems Ultrasonic, Inc. Model W-225R, Plain View, NY) set to a power level of 3 and a 30% duty cycle. The sonicator probe was immersed in the sample, and three to five pulses of sonication were used over ~5 seconds. The probe was washed between each sample with 10% bleach solution, rinsed with distilled water, and wiped dry with a kimwipe. Dummy samples without cells were sonicated and used as negative controls. Ribosomal DNA regions were amplified with the following primer combinations EukA-EukB (SSU), Dino1662F-25R1 (ITS and part of LSU) and 25F1-LSUR2 (last part of LSU) for '*Pheopolykrikos' hartmannii *(Table 3) [58-62]. The resulting products were sequenced with the same primers they were amplified with and the primers D3A and DLSUR2 (Table 3) [63] and assembled into a contig of 3,637 bases covering the SSU-ITS-LSU region. For the "*Warnowia *sp." (Florida), the EukA-EukB PCR product was used as a template for the following nested PCR amplifications: EukA-DinoR, SR4–SR9 and SR8–SR12 (Table 3) [58,59,61,63]; single PCR bands derived from each primer pair were sequenced with internal primers shown in Table 3. PCR experiments were run in 20-μL volumes with the following final concentrations: 500 mg/mL BSA (Sigma A2053), 50 mM Tris HCl (pH 8.3), 3 mM Mg, 10 μM dNTPs, 0.12 units of Promega Go-Taq, and 4 μL of sample (1 μL in the case of nested PCR). Cycling conditions were 95°C for 2 min, followed by 40 cycles of the following: 95°C for 30 s, 55°C for 30 s, 72°C for 1.5 min. This was followed with a 72°C step for 5 min after which the reactions were held at 4°C. The products from these reactions were visualized on ethidium bromide stained 1.5% agarose gels, precipitated using PEG (20% w/v polyethylene glycol, mw 8000, 2.5 M NaCl solution), washed with 70% ethanol, briefly air dried, resuspended in 10 μL of distilled water, and sequenced using Big Dye Terminator Cycle Sequencing Ready Reaction Kit (Applied Biosystem, Foster City, California) and an ABI 3730 sequencer. Sequence identity was evaluated initially by BLAST using the NCBI nonredundant database [64] and then by phylogenetic analyses.

### Alignments and molecular phylogenetic analyses

The new SSU and LSU rDNA sequences were aligned with other alveolate sequences using MacClade 4 [65], forming a 45-taxon and 36-taxon alignment respectively. However, we also analyzed our new LSU sequences within the context of several shorter-length sequences retrieved from GenBank, forming a 47-taxon alignment, and concatenated our SSU-LSU rDNA sequences where possible, forming a 17-taxon alignment. These four alignments are available on request. Maximum likelihood (ML) and Bayesian methods using the General Time Reversible (GTR) model of nucleotide substitutions were performed on all four alignments, this model was selected with MODELTEST version 3.06 [66]. All gaps were excluded from the alignments prior to phylogenetic analysis. The alpha shape parameters were estimated from the data using GTR, a gamma distribution with invariable sites and four rate categories (45-taxon SSU alignment with 1693 sites: ∝ = 0.378, i = 0.282; 36-taxon LSU alignment with 855 sites: ∝ = 0.509, i = 0.115; 47-taxon LSU alignment with 358 sites: ∝ = 0.742, i = 0.132; 17-taxon SSU-LSU alignment with 2549 sites: ∝ = 0.469, i = 0.339). ML trees analyzed using the parameters listed above were constructed with PhyML [67,68]. ML bootstrap analyses were also performed with PhyML (GTR+I+G model) on five hundred re-sampled datasets (one heuristic search per dataset) from each of the four alignments.

The four alignments were analyzed with Bayesian methods using the MrBayes program 3.1.2 [69,70]. The program was set to operate with a gamma distribution and four Monte-Carlo-Markov chains (MCMC) starting from a random tree. A total of 2,000,000 generations were calculated with trees sampled every 50 generations and with a prior burn-in of 100,000 generations (2000 sampled trees were discarded; burn-in was checked manually). A majority rule consensus tree was constructed from 38,001 post-burn-in trees. Posterior probabilities correspond to the frequency at which a given node was found in the post-burn-in trees. Independent Bayesian runs on each alignment yielded similar results.

## Results & Discussion

### Warnowiids evolved from within the Gymnodinium sensu stricto clade

**Figure 2:**
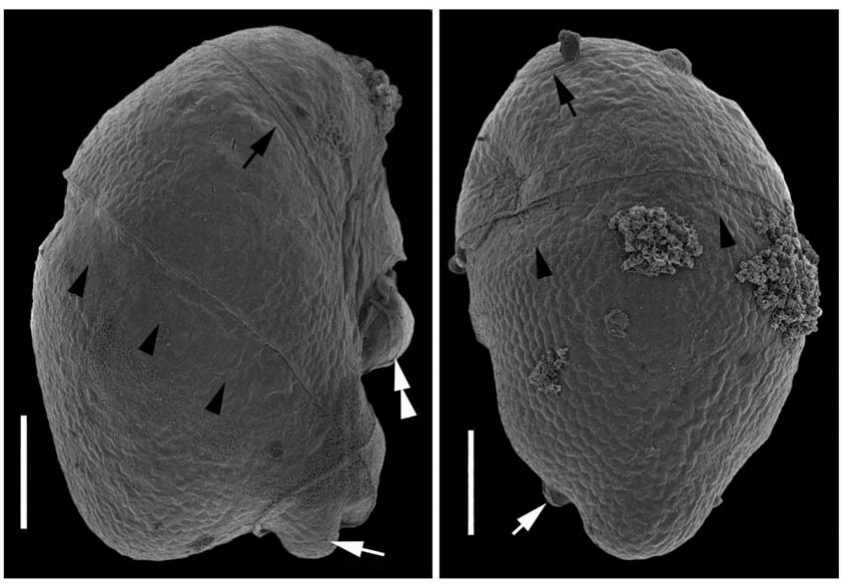
**Scanning electron micrographs of '*Proterythropsis*' sp. (British Columbia)**. These micrographs show a hundreds of small alveoli, a loop-shaped acrobase (black arrow), the cingulum (black arrowheads), a posterior cell 'extension' (white arrow), and the ocelloid (white double arrowheads). **(a) **Right lateral to ventral view **(b) **Dorsal view. Scale bars 10 μm.

**Figure 3:**
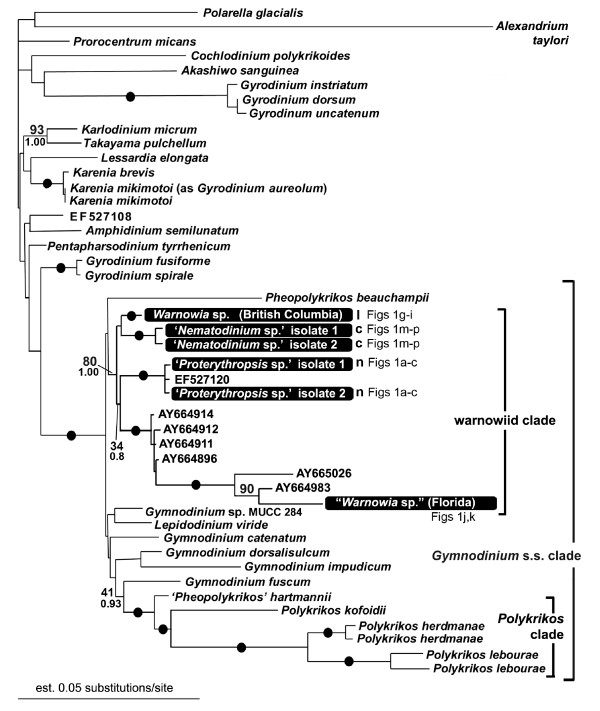
**SSU rDNA phylogeny**. Gamma-corrected maximum likelihood tree (-lnL = 8391.020304, α = 0.378, 4 rate categories) inferred using the GTR model of substitution on an alignment of 45 SSU rDNA sequences and 1693 unambiguously aligned sites. Numbers at the branches denote bootstrap percentages using maximum likelihood – GTR (top) and Bayesian posterior probabilities – GTR (bottom). Black dots on branches denote bootstrap percentages and posterior probabilities of 95% or higher. Accession numbers represent environmental sequences of unknown identity. Sequences derived from this study are highlighted in black boxes and the image/s in Fig 1 representing the taxon are cited. I = morphotype I of the genus *Warnowia*; c = containing chloroplasts; n = possessing nematocysts.

**Figure 4:**
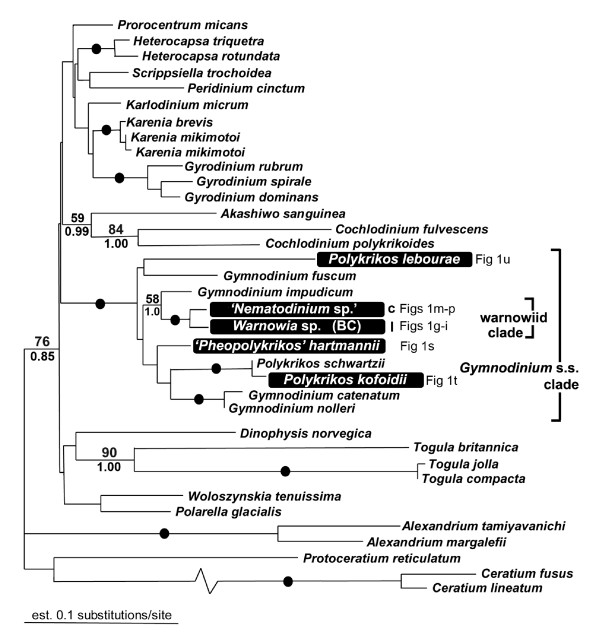
**LSU rDNA phylogeny**. Gamma-corrected maximum likelihood tree (-lnL = 8068.27939, α = 0.509, 4 rate categories) inferred using the GTR model of substitution on an alignment of 36 LSU rDNA sequences and 855 unambiguously aligned sites. Numbers at the branches denote bootstrap percentages using maximum likelihood – GTR (top) and Bayesian posterior probabilities – GTR (bottom). Black dots on branches denote bootstrap percentages and posterior probabilities of 95% or higher. Sequences derived from this study are highlighted in black boxes and the image/s in Fig 1 representing the taxon are cited. I = morphotype I of the genus *Warnowia*; c = containing chloroplasts.

The SSU- and LSU-rDNA sequences reported here were derived from cells like those shown in Figure 1. The general surface morphology of warnowiids, as represented by '*Proterythropsis*' sp. (British Columbia), consisted of many small alveoli, a loop-shaped acrobase, an obliquely oriented cingulum and an ocelloid (Figure 2). These morphological data are consistent with the only other known SEMs of warnowiids, namely *Erythropsidinium, Warnowia *and *Nematodinium *[24]. The phylogenetic position of the warnowiid sequences within the dinoflagellates is shown in the following figures: Figure 3 – the SSU rDNA alignment of 45 taxa and 1693 sites containing many athecate dinoflagellates and environmental sequences; Figure 4 – the LSU rDNA alignment of 36 taxa and 855 sites; Additional file 1 – the shorter-length LSU rDNA alignment of 47 taxa including only 358 sites; and Additional file 2 – the combined SSU- and LSU-rDNA alignment of 17 taxa and 2549 sites. These molecular phylogenetic data demonstrated that warnowiids form a moderately supported clade that falls within the *Gymnodinium *sensu stricto (s.s.) clade with very strong statistical support (Figures 3 and 4, Additional files 1 and 2). Although the nearest sister lineage to the warnowiid clade was unresolved in all of our analyses, the following taxa also clustered strongly within the *Gymnodinium *s.s.: *Pheopolykrikos beauchampii*, the *Polykrikos *clade and several species of *Gymnodinium *and *Lepidodinium *(e.g. *G. impudicum*, *G. fuscum*, *G. dorsalisulcum*, *G. catenatum*, *L. chlorophorum *and *L. viride*) (Figures 3 and 4, Additional files 1 and 2). Therefore, the *Gymnodinium s.s*. clade contains several well delimited genera in addition to *Gymnodinium *[26-28].

The phylogenetic relationships within the warnowiid clade were poorly resolved near the backbone (Figure 3). However, the molecular phylogenetic analyses did demonstrate that the two isolates of '*Nematodinium*' grouped strongly together, and this clade formed the nearest sister group to the *Warnowia *sp. collected from British Columbia (Figures 3 and 4, Additional files 1 and 2). The two isolates of '*Proterythropsis*' also grouped strongly together with an environmental sequence, namely EF527120, collected from Framvaren Fjord, Norway, (Figure 3) [A. Behnke, pers. comm.]. The "*Warnowia *sp." collected from Florida was much more divergent and clustered strongly with several environmental sequences collected from Sargasso Sea (Figure 3) [Armbrust et al., unpublished]; two of the environmental sequences, namely AY664983 and AY665026, formed the nearest sister lineages to this "*Warnowia *sp." (Florida) (Figure 3). The molecular phylogenetic analyses of SSU rDNA also indicated that the following environmental sequences probably represent a single, potentially undescribed, warnowiid species: AY664914, AY664912, AY664911 and AY664896 (Figure 3). The generated SSU rDNA sequence for *Erythropsidinium *was too divergent to be included into the analysis.

Daugbjerg et al. [10] hypothesized that the warnowiid *Nematodinium armatum *is related to the *Gymnodinium s.s*., because of the presence of a nuclear fibrous connector and the loop-shaped acrobase. This is the only published hypothesis about the possible relationship of a warnowiid taxon to other dinoflagellate taxa, and our molecular phylogenetic analyses reinforced this hypothesis. The characteristic feature for the *Gymnodinium s.s*. clade is the loop-shaped acrobase which has been demonstrated in the *Gymnodinium *species within this clade, *Lepidodinium*, '*Pheopolykrikos*' *hartmannii*, *Polykrikos *spp., and *Nematodinium armatum *[e.g., [24,26-28]]. We have shown for the first time that a loop-shaped acrobase is also present in '*Proterythropsis*' sp. (Figure 2) and hypothesize, based on our molecular phylogenetic data, that a similar acrobase morphology is present in the *'Nematodinium' *and *Warnowia *species isolated from British Columbia (Figure 3).

We present the first LSU rDNA sequence for *P. lebourae*. Although a strongly supported *Polykrikos *clade, within the *Gymnodinium s.s*. clade, was shown previously in SSU phylogenies [27,28] (Figure 3), this clade did not receive strong statistical support in our phylogenetic analyses of LSU rDNA sequences (Figure 4 and Additional file 1) [71].

### Character evolution in warnowiids

**Figure 5:**
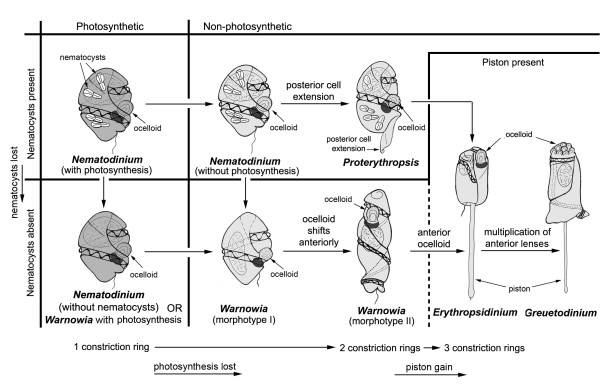
**Hypothetic framework(s) for understanding character evolution in warnowiid dinoflagellates as inferred from known morphological diversity and the molecular phylogenetic results of this study (partly after Greuet 1978)**. Darker grey cells are photosynthetic. Characters of interest are parsimoniously mapped onto the framework. Arrows refer to possible trajectories of character state lossess and transformations; where phylogenetic relationships are unknown, more than one possible transformation is indicated (e.g. the origin of pistons and anterior ocelloids from either a *Proterythropsis*-like ancestor or a *Warnowia *morphotype II-like ancestor).

The presence of nematocysts in both warnowiids and polykrikoids suggests that the most recent common ancestor of both lineages already possessed these complex organelles [28]. This hypothesis would be significantly bolstered if it were demonstrated in molecular phylogenetic analyses that the two clades are indeed closely related to one another. Our results strongly demonstrated that warnowiids and polykrikoids are both members of the *Gymnodinium s.s*. clade; however, a specific sister relationship between the two subgroups, to the exclusion of *Gymnodinium *and *Lepidodinium *sequences, remained unresolved in all four datasets (Figures 3 and 4, Additional files 1 and 2). Regardless, when considering the overall phylogenetic distribution of nematocysts within the *Gymnodinium s.s*. clade, the most parsimonious explanation requires that nematocysts originated once in the most recent ancestor of polykrikoids and warnowiids and were subsequently lost, at least once, within the warnowiid clade (Figure 5). Although less parsimonious scenarios can also explain the distribution of nematocysts in polykrikoids and warnowiids (e.g., several independent gains or kleptocnidae), there is currently no evidence to support these alternatives.

The ocelloid is perhaps the most striking feature of all dinoflagellates and is the best synapomorphy for the warnowiid clade as inferred from SSU- and LSU-rDNA sequences (Figures 3, 4 and 5). Greuet [42] performed the pioneering ultrastructural research on warnowiids, and the structural diversity found in different ocelloids led him to formulate a hypothesis about character evolution within the group; variations of ocelloids have been described in *Nematodinium*, '*Warnowia *morphotype II', and *Erythropsidinium *[35,42-48]. Greuet [42] hypothesized that during the evolution of warnowiids, the ocelloid increased in complexity:

(1) the number of (iris-like) constriction rings increased from 1 → 2 → 3, (2) the ocelloid increased in size, and (3) the position of the ocelloid gradually shifted toward the anterior end of the cell (Table 2, Figure 5). We have extended this hypothesis by incorporating the following events: (4) the presence of nematocysts is an ancestral state that was subsequently lost in more derived lineages, (5) the piston was gained in the most recent ancestor of *Erythropsidinium *and *Greuetodinium*, and (6) the lense region or 'hyalosome' of the ocelloid was multiplied in the most recent ancestor of *Greuetodinium *(Figure 5). Moreover, because *Nematodinium armatum *contains nematocysts and is the only known photosynthetic species, we hypothesize that photosynthesis involving typical peridinin-containing dinoflagellate plastids is an ancestral state for warnowiids that was subsequently lost early in the history of the clade (Figure 5). Mornin and Francis [35] wrote that the plastids of *N. armatum *lack thylakoids, which would preclude photosynthesis; however, one of the TEM images published in this study (namely, Figure "a" on plate III) shows a plastid with many thylakoids. Because the relatively low magnification of this image does not allow us to determine whether the plastids have the usual dinoflagellate morphology (i.e., thylakoids in stacks of three and three outer membranes), an ultrastructural reinvestigation of this species is needed.

The species from which we were able to acquire SSU- and LSU-rDNA sequences represent only a part of the morphological diversity found in warnowiid dinoflagellates. All of the species we examined possessed an ocelloid located near the posterior end of the cell, which is inferred to be an ancestral state for the group. However, one species, namely '*Proterythropsis*' sp., possessed nematocysts, and one species, namely '*Nematodinium*' sp., was photosynthetic. Interestingly, the taxa without nematocysts, namely '*Nematodinium*' sp. and *Warnowia *sp. (British Columbia), clustered together in the phylogenies inferred from SSU rDNA sequences, albeit with weak support (Figure 3); these taxa also clustered together in the phylogeneis inferred from LSU rDNA sequences, but they were the only two warnowiid species in the analyses (Figure 4 and Additional files 1 and 2). The "*Warnowia *sp." (Florida) showed the most divergent position in the phylogenies inferred from SSU rDNA sequences and clustered strongly with environmental sequences (Figure 3). Although all of these results are consistent with the hypothetical framework(s) shown in Figure 5, SSU- and LSU-rDNA sequences from relatively scarce planktonic warnowiids – like *Warnowia *morphotype II, *Erythropsidinium*, and *Greuetodinium *– will be required to more comprehensively evaluate character evolution within the group.

### Taxonomic considerations

A survey of the literature on warnowiids reveals several taxonomic problems associated with the genera *Warnowia*, *Nematodinium*, and *Proterythropsis *(Table 2). For instance, *Warnowia *currently contains two very different morphotypes with respect to the cingulum, sulcus, acrobase(s), and the position of the ocelloid [24,29,36,37] (Table 2). Species in the genus are characterized by the absence of features, such as plastids, nematocysts, pistons, and posterior cell extensions (Table 2). *Warnowia *morphotype I is similar to *Nematodinium armatum*, and the cells of *Warnowia *morphotype II are distinctively elongated (Figure 5). *Nematodinium *species also show a wide range of cingulum and sulcus morphologies, but are defined by the presence of nematocysts and a posterior ocelloid and the absence of a posterior cell extension (Table 2). However, Hulburt [49] reported specimens of *Nematodinium armatum *without nematocysts. These cells also fit the circumscription of *Warnowia *type I, but they are photosynthetic [34,35,49]. The genus *Proterythropsis *is recognized by the presence of a posterior cell extension and is circumscribed as having a posterior ocelloid and nematocysts (Table 2, Figure 5) [36,38]; however, Kofoid and Swezy [36] reported *Proterythropsis *cells without nematocysts. Therefore, it is unclear whether nematocysts are a stable taxonomic character at any level in the phylogenetic hierarchy. In fact, it is not even clear whether all specimens of a species possess nematocysts during all stages of their life cycle or whether there are situations when nematocysts are simply unrecognizable with light microscopy during some developmental stages [e.g., [29]].

Species concepts within warnowiids are also highly problematic. It has been shown that the structure, color and position of the ocelloid can change during the course of cell development and division [37] (also see our Figures 1a and 1d of the same species). Because of this variability, many previously described species probably represent conspecifics [e.g., [36-38]].

These taxonomic ambiguities have made the identification of warnowiid genera and species extremely challenging for us when isolating cells from natural samples. Therefore, in this study, we have decided not to name the taxa to the species level and to demarcate ambiguous genus assignments with quotation marks. Our '*Proterythropsis*' sp. has a relatively short posterior cell 'extension' and nematocysts (Figures 1a, b, c, d, e and 1f); it essentially possesses morphological features that are intermediate between *Proterythropsis *sensu stricto and heterotrophic *Nematodinium *cells. Although our '*Nematodinium*' sp. looks most like *Nematodinium armatum*, we were unable to detect nematocysts (Figures 1m, n, o and 1p). It is possible that this species best represents an undescribed photosynthetic version of *Warnowia *morphotype I; however, because these cells could also represent *Nematodinium armatum *without detectible nematocysts, as observed by Hulburt [49], we decided to tentatively assign this species to *Nematodinium*. The "*Warnowia *sp." (Florida) (Figures 1j and 1k) possessed morphological features that were intermediate between morphotypes I and II, but we are not able to confirm the presence or absence of nematocysts; only one specimen was available for our investigations. The *Warnowia *sp. (British Columbia) possessed the morphological features of morphotype I (Figures 1g, h and 1i) and divided within hyaline cysts. The genus *Erythropsidinium *was the easiest to identify due to the conspicuous piston and the anterior position of the ocelloid (Figures 1q and 1r).

### Warnowiid environmental sequences

The DNA sequences we report here demonstrate that warnowiid dinoflagellates have been unknowingly recorded in previous environmental PCR surveys of biodiversity (Figure 3, accession numbers represent environmental sequences of uncultured eukaryotes with unknown morphology/identity). '*Proterythropsis*' sp. or a very close relative (EF527120, Figure 2) was sequenced/detected in a sample from Framvaren Fjord, Norway, in September 2005 (A. Behnke, pers. comm.). The sampled water was anoxic with measurable H_2_S (unpublished data, A. Behnke, pers. comm.). To the best of our knowledge, this is the first report of a warnowiid from anoxic habitats. Relatives of "*Warnowia *sp." (Florida) were sequenced from nanoplankton samples taken from Sargasso Sea eddies (Armbrust et al. unpublished, from GenBank, acc. no. AY664983 and AY665026). Four additional sequences of that survey (Armbrust et al. unpublished, from GenBank, acc. no. AY664911, AY664912, AY664914, and AY664896) clustered together as sister lineage(s) to the "*Warnowia *sp." (Florida) clade (Figure 3). How warnowiids could be part of the nanoplankton fraction of a water sample is difficult to explain with present data and might indicate a more complex lifecycle involving nanoflagellated stages; all described species of warnowiids belong to the microplankton (> 20 μm). Contamination of the nanoplankton sample with free-floating DNA from ruptured microplanktonic organisms could also explain these findings. Nonetheless, the warnowiid environmental sequences demonstrate previously undetected diversity in the group and the sequences that we report here help provide cellular identities to these clades.

## Conclusion

This study reports the first molecular phylogenetic data from uncultivated warnowiid dinoflagellates collected from both the Pacific and Atlantic Oceans, namely SSU- and LSU-rDNA sequences from probably three different genera of warnowiids (*'Nematodinium'*, *Warnowia *and *'Proterythropsis'*), and partial LSU-rDNA sequences from three different species of polykrikoids (*P. kofoidii*, *P. lebourae*, and *'Pheopolykrikos' hartmannii*). All of the investigated species clustered within the *Gymnodinium sensu stricto *(*s.s*.) clade with very strong support, which was concordant with comparative morphological data. The warnowiid sequences clustered together in one well-supported clade, which reinforced the ocelloid as the best synapomorphy for this group. Comparative morphological data and molecular phylogenetic data demonstrate that the polykrikoid clade and the warnowiid clade are closely related to each other, but the precise branching order within the *Gymnodinium *s.s. clade remains unresolved. Nonetheless, the most parsimonious scenario of character evolution suggests that the most recent common ancestor of polykrikoids and warnowiids possessed nematocysts, and probably photosynthetic plastids, that were subsequently lost during the early evolution of the warnowiid clade. Species and genus concepts in warnowiids are highly problematic and a comprehensive taxonomic revision is needed in order to better understand the evolutionary history of the group. However, additional molecular and morphological data is severely hindered by the extraordinary rarity that these planktonic dinoflagellates are encountered in natural samples. Accordingly, this study represents a first step toward meeting these aims and provides a set of preliminary DNA barcodes for warnowiids that not only helps advance the systematics of the group, but also improves inferences about the evolutionary history that gave rise to some of the most sophisticated organelles ever discovered in eukaryotic cells: ocelloids.

## List of abbreviations

GTR model: general time-reversable model; HKY model: Hasegawa-Kishino-Yano model; LSU rDNA: large subunit ribosomal DNA; MCMC: Monte-Carlo-Markov chains; ML: maximum likelihood; PCR: polymerase chain reaction; SSU rDNA: small subunit ribosomal DNA.

## Authors' contributions

MH did the collection, identification, photographing, and sequencing of dinoflagellate taxa from British Columbia, Canada; reviewed the literature, formulated the hypothesis, wrote the first draft of the manuscript and assembled the Figures.

TRB and SMH participated in the collection, identification, photographing, and sequencing of samples taken from Maryland and Florida, USA.

CFD supervised the collection from FL, helped with photography, data analysis, and funded the collections from FL and MD.

BSL funded and supervised the collection of data from British Columbia, Canada; constructed the multiple sequence alignments; performed the phylogenetic analyses and SEM work; and helped write and edit the manuscript.

All authors have read and approved the final manuscript.

## Supplementary Material

Additional file 1 **LSU rDNA phylogeny**. Gamma-corrected maximum likelihood tree (-lnL = 4115.21736, α = 0.742, 4 rate categories) inferred using the GTR model of substitution on an alignment of 47 LSU rDNA sequences and 358 unambiguously aligned sites. Numbers at the branches denote bootstrap percentages using maximum likelihood – GTR (top) and Bayesian posterior probabilities – GTR (bottom). Black dots on branches denote bootstrap percentages and posterior probabilities of 95% or higher. Sequences derived from this study are highlighted in black boxes. Click here for file

Additional file 2 **Concatenated SSU and LSU rDNA phylogeny**. Gamma-corrected maximum likelihood tree (-lnL = 10274.67788, α = 0.496, 4 rate categories) inferred using the GTR model of substitution on an alignment of 17 combined SSU and LSU rDNA sequences and 2549 unambiguously aligned sites. Numbers at the branches denote bootstrap percentages using maximum likelihood – GTR (top) and Bayesian posterior probabilities – GTR (bottom). Black dots on branches denote bootstrap percentages and posterior probabilities of 95% or higher. Sequences derived from this study are highlighted in black boxes. Click here for file
